# Lipoprotein(a) in Personalized Cardiovascular Prevention: Risk Stratification, Coronary Artery Calcium, and Emerging Lp(a)-Lowering Therapies

**DOI:** 10.3390/jcm15176640

**Published:** 2026-08-28

**Authors:** Alveena Batool Syed, Samuel Michalak, Indra Kiran Reddy Potulapati, Aravinthan Vignarajah, Gautam V. Shah

**Affiliations:** 1 Department of Internal Medicine & Geriatrics, Cleveland Clinic Foundation, Cleveland, OH 44111, USA; 2 Integrated Hospital Care Institute, Cleveland Clinic Foundation, Cleveland, OH 44195, USA; 3 Heart Vascular & Thoracic Institute, Cleveland Clinic Foundation, Cleveland, OH 44195, USA; shahg@ccf.org

**Keywords:** lipoprotein(a), Lp(a), dyslipidemia guideline, ASCVD risk, PREVENT, coronary artery calcium, PCSK9 inhibitors, antisense oligonucleotide, siRNA, apheresis

## Abstract

Lipoprotein(a) [Lp(a)] is an independent and causal risk factor for atherosclerotic cardiovascular disease (ASCVD) and calcific aortic valve stenosis (CAVS), with plasma levels largely genetically determined and minimally influenced by lifestyle. Contemporary dyslipidemia guidelines now recommend at least one lifetime measurement and increasingly incorporate Lp(a) into personalized cardiovascular risk evaluation. Recent guideline updates integrate traditional risk estimation with risk-enhancing factors and coronary artery calcium (CAC) imaging. The 2026 U.S. multi-society guideline classifies Lp(a) ≥ 125 nmol/L as a risk-enhancing factor and ≥250 nmol/L as a major risk enhancer. The PREVENT equations provide baseline risk estimation, personalized by Lp(a) and refined through CAC-based reclassification when treatment decisions remain uncertain. Despite its established role in risk assessment, current management remains indirect and emphasizes aggressive control of modifiable risk factors, particularly low-density lipoprotein cholesterol (LDL-C). Emerging Lp(a)-targeted therapies, including antisense oligonucleotides, small interfering RNA agents, and oral small-molecule inhibitors, have shown substantial and sustained reductions in Lp(a) levels, although definitive evidence of cardiovascular benefit is still pending. This review provides a practical, evidence-based synthesis of Lp(a) biology, contemporary guideline recommendations, integrated risk stratification using PREVENT and CAC, current management, and emerging Lp(a)-targeted therapies for use across primary care and cardiology practice.

## 1. Introduction

**Figure 1:**
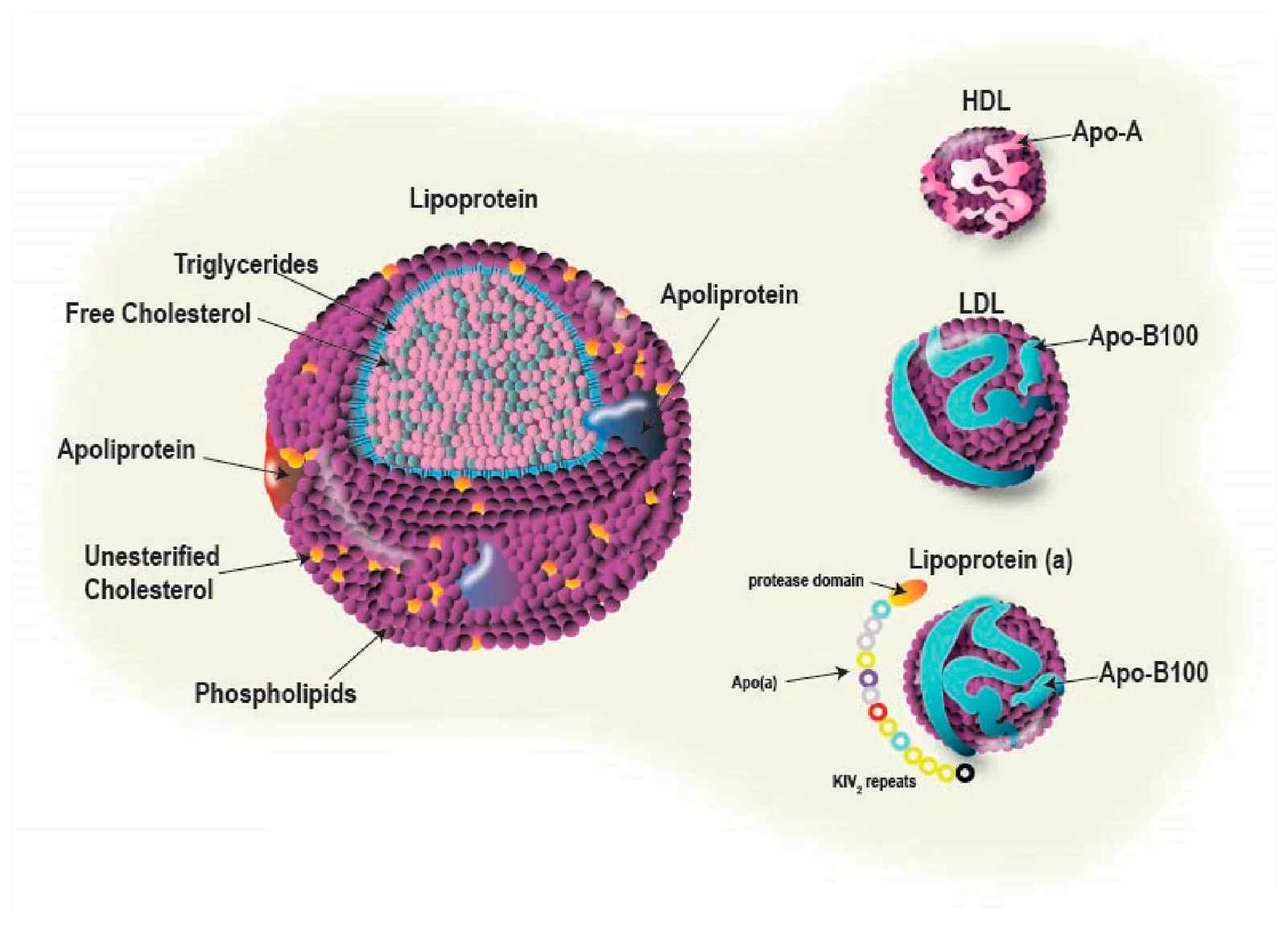
Unique structure of Lp(a), highlighting the Apo(a) kringle domains and their role in pathogenicity. The colored circles represent the different kringle IV (KIV) domains of apo(a), including the variably repeated KIV type 2 domains, while the protease-like domain is shown separately. Apo(a) is covalently linked to apolipoprotein B-100 (apoB-100) of an LDL-like particle.

Lipoprotein(a) [Lp(a)] is a low-density lipoprotein (LDL)-like particle that has gained significant attention as an independent causal risk factor for atherosclerotic cardiovascular disease (ASCVD) and calcific aortic valve stenosis [1,2]. It consists of an LDL particle linked to apolipoprotein(a), a distinctive protein that confers atherogenic, prothrombotic, and inflammatory properties [1,3]. Apolipoprotein(a) is composed of structural domains known as kringles [Figure 1]. Polymorphism in the kringle IV type 2 repeat region of the LPA gene results in Lp(a) particles of varying sizes; smaller apolipoprotein(a) isoforms are associated with higher plasma Lp(a) concentrations. Although Lp(a) has no clearly established physiological role, it is a major cardiovascular risk factor. Globally, approximately 20–25% of individuals have elevated Lp(a) levels (commonly defined as >125 nmol/L). Elevated Lp(a) concentrations are largely genetically determined, remain relatively stable after early childhood, and are minimally influenced by lifestyle or environmental factors. Individuals of African ancestry have significantly higher median Lp(a) levels than those of European or Asian descent [1,2,3,4] (Figure 1).

## 2. Methods

This narrative review was informed by a structured literature search of MEDLINE/PubMed, Embase, and Scopus, supplemented by searches of ClinicalTrials.gov and relevant professional-society guideline repositories. Search concepts included “lipoprotein(a),” “Lp(a),” “cardiovascular risk,” “atherosclerotic cardiovascular disease,” “aortic stenosis,” “PREVENT,” “coronary artery calcium,” “risk stratification,” “lipoprotein apheresis,” “pelacarsen,” “olpasiran,” “lepodisiran,” “zerlasiran,” “siRNA,” “antisense oligonucleotide,” and “muvalaplin.” Contemporary guidelines, consensus statements, randomized and prospective clinical studies, major observational studies, and registered clinical trials relevant to Lp(a) measurement, risk assessment, clinical management, and emerging therapies were prioritized. Reference lists of major consensus documents and pivotal studies were also reviewed. Evidence selection was based on clinical relevance, methodological quality, and relevance to the scope of this narrative review. ClinicalTrials.gov was used to verify the status and design of ongoing investigational studies. The literature search was updated through May 2026.

## 3. Lp(a) as an Independent Risk Factor for CVD

A substantial body of evidence establishes lipoprotein(a) [Lp(a)] as an independent contributor to cardiovascular risk [2,5]. Large epidemiological studies and Mendelian randomization analyses involving hundreds of thousands of patients demonstrate a strong, continuous association between plasma Lp(a) concentrations and the risk of atherosclerotic cardiovascular disease (ASCVD). This relationship appears causal, as genetic variants in the LPA gene that increase Lp(a) levels are associated with increased rates of myocardial infarction and coronary artery disease. Elevated Lp(a) is consistently associated with a higher risk of coronary heart disease and ischemic stroke across diverse populations, independent of traditional risk factors. Notably, Lp(a) confers excess risk even among individuals with otherwise optimal LDL cholesterol, underscoring that it represents a distinct and additive atherogenic pathway [1,2,3,4,5,6,7].

Elevated Lp(a) is also strongly implicated in calcific aortic valve disease. Prospective cohort studies and genetic analyses support a causal role for aortic valve calcification in the progression to aortic stenosis. Mechanistically, apolipoprotein(a) preferentially accumulates in valvular tissue and transports pro-calcific oxidized phospholipids, promoting inflammation and mineralization.

In contrast, Lp(a) does not appear to be a major risk factor for venous thromboembolism, as studies have not consistently demonstrated an association with deep vein thrombosis or pulmonary embolism. Collectively, the weight of evidence, supported by genetic studies and expert consensus, establishes elevated Lp(a) as an independent, genetically mediated risk factor for ASCVD and calcific aortic valve disease [1,2,3,4,5,6,7].

## 4. Pathophysiological Mechanisms

Several mechanisms explain how Lp(a) promotes atherosclerosis and thrombosis. Structurally, apolipoprotein(a) is highly homologous to plasminogen, the precursor of the fibrinolytic enzyme plasmin. Apo(a) shares substantial structural homology with plasminogen, which has led to proposed mechanisms involving interference with fibrinolysis and other thrombosis-related pathways. Although these mechanisms are biologically plausible and supported by experimental observations, their clinical significance remains less firmly established than the atherogenic and inflammatory effects of Lp(a), particularly because epidemiologic studies have not demonstrated a consistent association between elevated Lp(a) and venous thromboembolism [1,2,3].

In addition, Lp(a) particles are enriched in oxidized phospholipids (OxPL), which are present both within the LDL-like core and covalently bound to apolipoprotein(a). Oxidized phospholipids are potent mediators of vascular inflammation and can promote monocyte activation, endothelial dysfunction, and pro-inflammatory signaling within the arterial wall, contributing to plaque formation and the development of unstable atherosclerotic lesions [7]. Like LDL, Lp(a) deposition within the arterial intima promotes cholesterol accumulation and foam cell formation, while its OxPL content amplifies chronic vascular inflammation. In calcific aortic valves, Lp(a) co-localizes with OxPL in areas of active calcification, supporting a role in the propagation of valvular disease. The combined atherogenic and inflammatory effects of Lp(a) likely underline its strong association with cardiovascular events [7,8,9].

**Figure 2:**
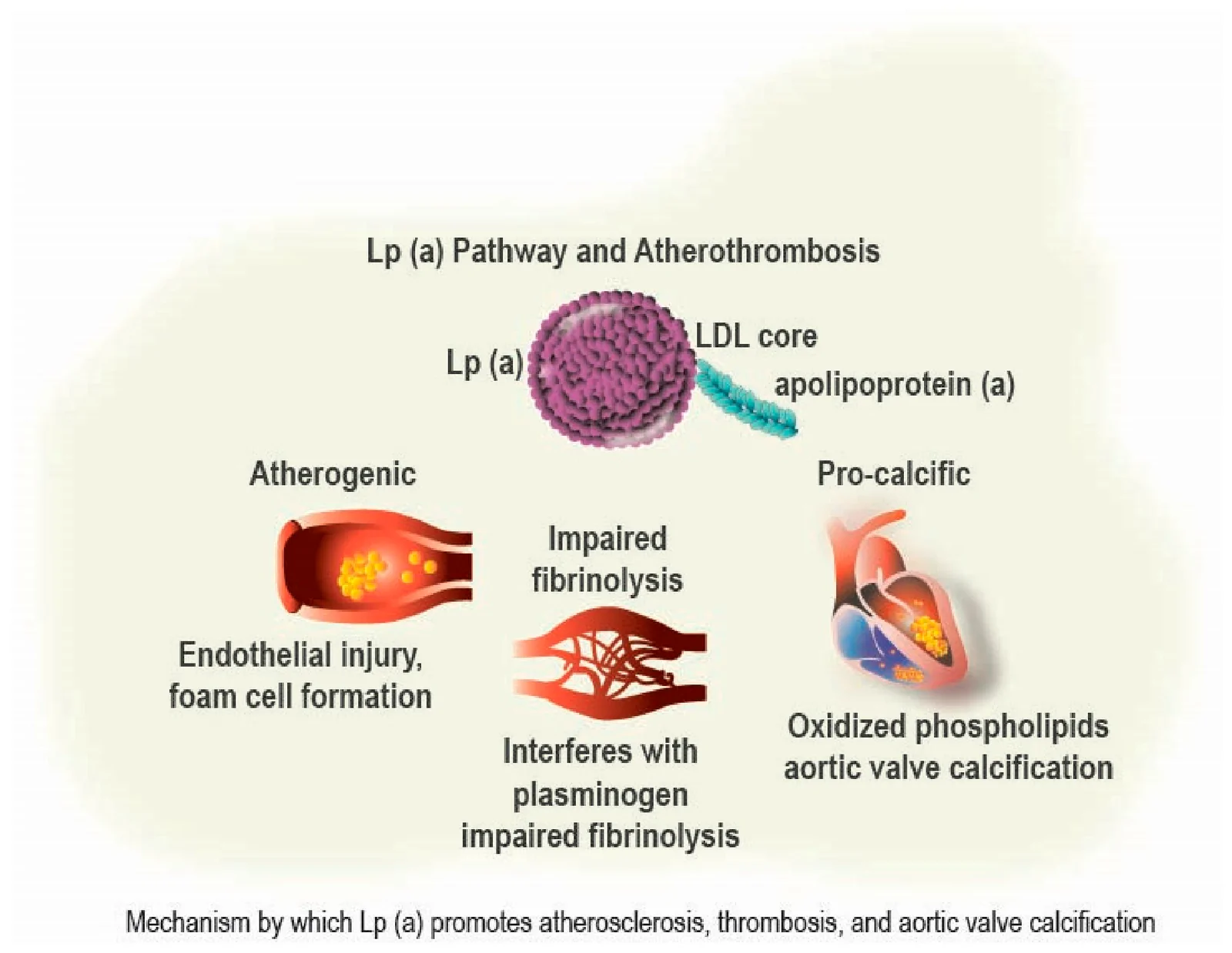
Pathophysiologic mechanisms linking lipoprotein(a) to atherothrombosis and aortic valve calcification.

**Figure 3:**
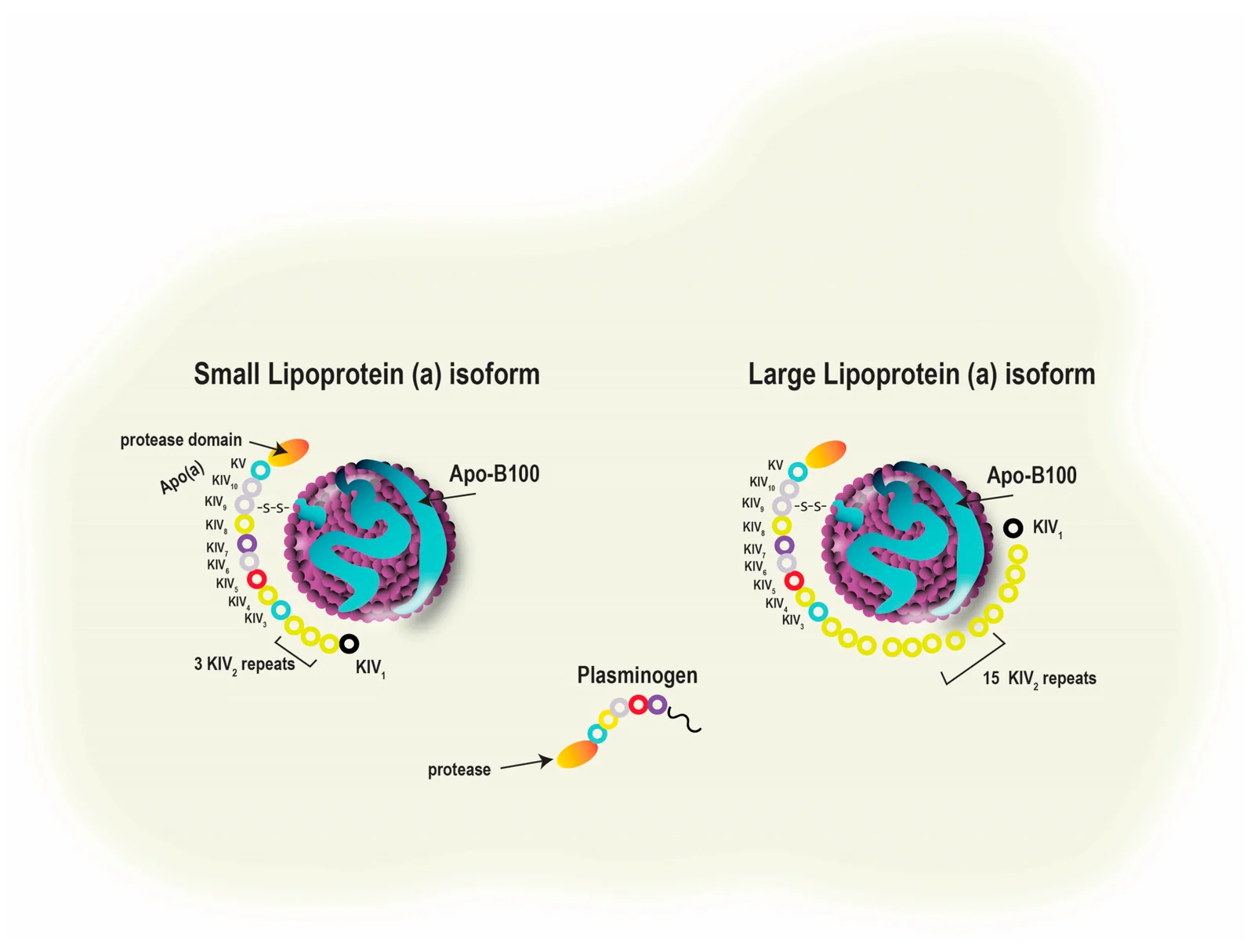
Structural heterogeneity of lipoprotein(a) and relationship of apolipoprotein(a) to plasminogen. Apo(a) contains multiple kringle domains homologous to plasminogen and exhibits substantial size variability, primarily determined by the number of kringle IV type 2 (KIV2) repeats. Smaller apo(a) isoforms are generally associated with higher circulating Lp(a) concentrations.

In summary, elevated Lp(a) creates a pro-atherogenic, pro-inflammatory, and prothrombotic milieu, contributing to increased risk of myocardial infarction, stroke, and atherosclerotic cardiovascular disease [7,8,9] (Figure 2 and Figure 3, Table 1).

## 5. Guideline and Consensus Recommendations on Lp(a)

Contemporary cardiovascular prevention guidelines increasingly recognize Lp(a) as an important component of risk assessment. Despite differences in terminology and threshold definitions, the 2026 ACC/AHA multi-society guideline, 2025 ESC/EAS Focused Update, 2024 NLA Focused Update, and 2022 EAS consensus broadly converge on once-in-adulthood measurement and interpretation of Lp(a) within an individual’s overall cardiovascular risk [10,11,12,13].

### 5.1. The U.S. 2026 Multi-Society Guideline (ACC/AHA)

The guideline and its official summary emphasize that lipoprotein(a) [Lp(a)] should be measured at least once in adulthood to identify individuals at higher risk of ASCVD [10]. Lp(a) ≥ 125 nmol/L or 50 mg/dL is considered a risk-enhancing factor and is associated with approximately a 1.4-fold higher long-term risk of myocardial infarction or stroke, while levels ≥ 250 nmol/L are associated with at least a twofold higher long-term risk. Within the guideline’s risk assessment framework, the PREVENT score is used to estimate baseline cardiovascular risk, after which Lp(a) is incorporated as a risk-enhancing factor to personalize risk assessment. Elevated Lp(a) should be considered as a risk-enhancing factor during clinician–patient risk assessment rather than as a validated coefficient for mathematically recalibrating an individual’s PREVENT-ASCVD estimate. In this context, Lp(a) serves as a trigger for more intensive LDL-C lowering and optimization of other risk factors. When uncertainty in risk stratification persists, selective use of coronary artery calcium (CAC) scoring is recommended for reclassification within the broader “Calculate–Personalize–Reclassify (CPR)” model [10,11].

### 5.2. The 2025 ESC/EAS Focused Update

The focused update recognizes a continuous increase in cardiovascular risk across the Lp(a) spectrum and supports measuring Lp(a) at least once in every adult’s lifetime. Lp(a) levels > 50 mg/dL or 100–125 nmol/L are considered a risk-enhancing factor (risk modifier) to refine cardiovascular risk estimation, particularly in individuals at moderate risk or near treatment decision thresholds. The document emphasizes the importance of assay standardization, noting variability related to apolipoprotein(a) isoform size and kringle repeat number, and recommends reporting in nmol/L when possible, although mg/dL remains acceptable in clinical practice. Lp(a) levels may increase after menopause, and repeat measurement can be considered in selected individuals when earlier values were borderline [12].

### 5.3. The 2024 National Lipid Association (NLA)

The NLA update recommends measuring lipoprotein(a) [Lp(a)] at least once in all adults and emphasizes that Lp(a)-associated risk spans a continuous spectrum rather than a single threshold. It proposes pragmatic clinical categories for interpretation: low (<75 nmol/L; approximately <30 mg/dL), intermediate (75–125 nmol/L; ~30–50 mg/dL), and high (≥125 nmol/L; ≥50 mg/dL). The statement endorses cascade screening of first-degree relatives of individuals with elevated Lp(a) and highlights the importance of intensified risk factor management, particularly aggressive LDL-C lowering, in those with elevated levels. In select high-risk patients, particularly those with familial hypercholesterolemia and established ASCVD, lipoprotein apheresis may be considered when LDL-C remains ≥100 mg/dL despite maximally tolerated therapy, especially when Lp(a) is markedly elevated [13].

### 5.4. The 2022 EAS Consensus Statement

The 2022 EAS consensus recommends measuring Lp(a) at least once in adulthood and interpreting results in the context of absolute global cardiovascular risk, with early, intensive management of modifiable risk factors for those with elevated Lp(a). It recognizes Lp(a) as causal for ASCVD and as causal or likely causal for aortic valve stenosis, supports implementation through care models potentially modeled on familial hypercholesterolemia programs, including cascade testing of relatives, and emphasizes the need for improved assay standardization and definitive outcomes trials of targeted Lp(a)-lowering therapies [1].

### 5.5. Comparative Synthesis

**Figure 4:**
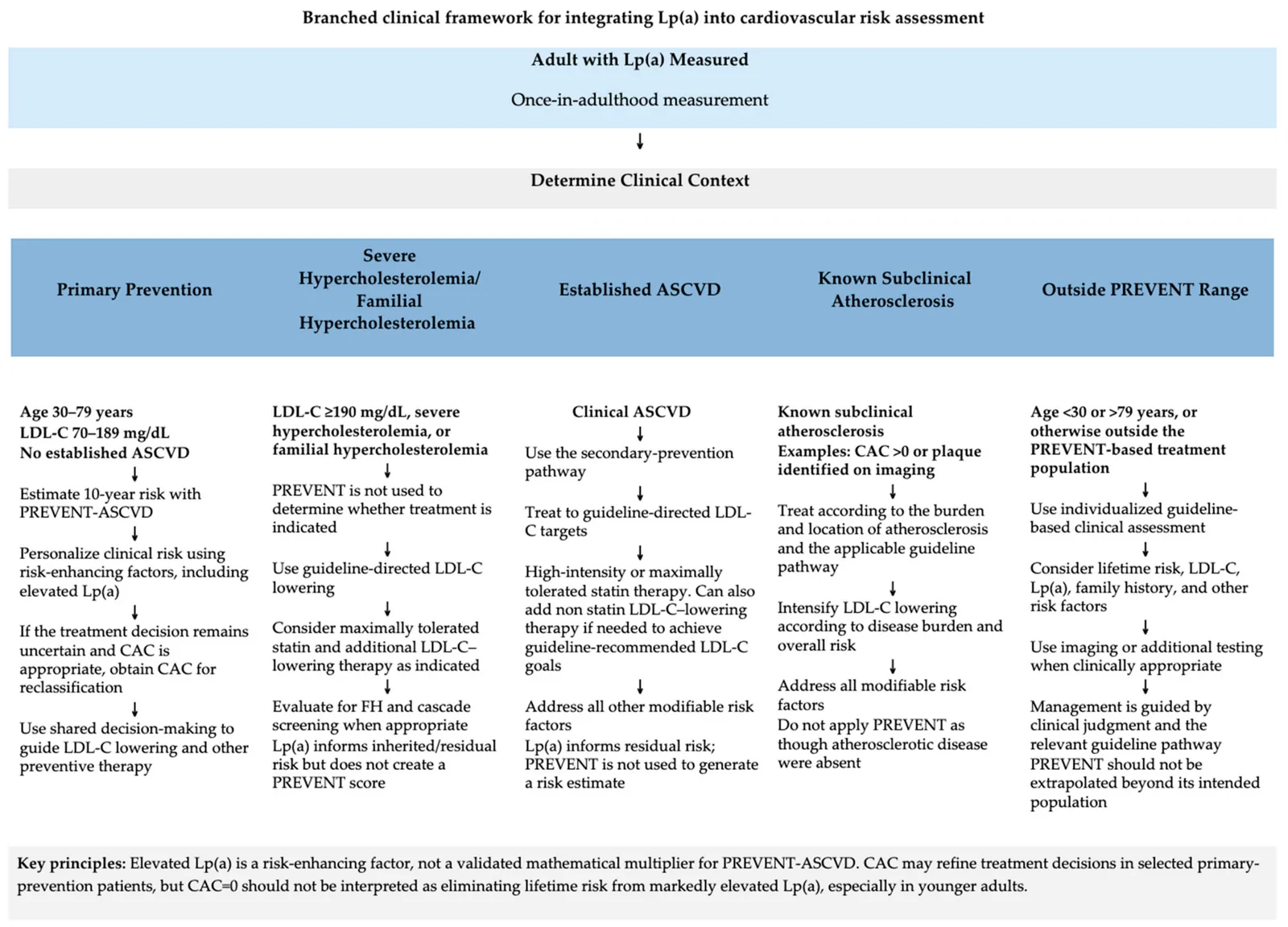
Branched framework for incorporating Lp(a) into cardiovascular risk assessment according to clinical context. PREVENT-ASCVD is used only within its intended primary-prevention population; patients with established ASCVD, severe hypercholesterolemia/familial hypercholesterolemia, known subclinical atherosclerosis, or those outside the PREVENT range should follow the corresponding guideline-based pathway. ASCVD, atherosclerotic cardiovascular disease; CAC, coronary artery calcium; FH, familial hypercholesterolemia; LDL-C, low-density lipoprotein cholesterol; Lp(a), lipoprotein(a); PREVENT, Predicting Risk of Cardiovascular Disease Events.

Across all four documents, there is strong convergence on several core principles: lipoprotein(a) [Lp(a)] should be measured at least once in adulthood; elevated Lp(a) confers continuous rather than binary risk; results should be interpreted in the context of overall absolute cardiovascular risk; and, in the absence of approved Lp(a)-specific outcome-proven therapies, management should focus on early, intensive control of modifiable risk factors, especially LDL-C lowering. Although there is strong consensus across societies regarding the clinical importance of Lp(a), differences primarily relate to terminology, threshold framing, and implementation within risk assessment frameworks. The 2022 EAS consensus is the most pathobiology- and systems-oriented document, emphasizing causality for ASCVD and aortic valve stenosis, global risk interpretation, assay standardization, cascade testing, and implementation models based on familial hypercholesterolemia care pathways. The 2024 NLA update is the most operationally pragmatic, introducing low (<75 nmol/L), intermediate (75–125 nmol/L), and high (≥125 nmol/L) clinical risk categories and explicitly discouraging fixed conversions between mg/dL and nmol/L because of apo(a) isoform heterogeneity. The 2025 ESC/EAS focused update places Lp(a) most explicitly within European risk refinement, describing >50 mg/dL or >105 nmol/L as a cardiovascular risk-enhancing factor/risk modifier and using it to refine treatment decisions, especially near decision thresholds [12]. The 2026 U.S. multi-society ACC/AHA guideline similarly treats Lp(a) ≥ 125 nmol/L (50 mg/dL) as a risk-enhancing factor but integrates it more explicitly into a structured prevention framework in which PREVENT estimates baseline risk, risk enhancers personalize that estimate, and selective CAC is used for reclassification within the Calculate–Personalize–Reclassify (CPR) model. Thus, the main differences are less about whether Lp(a) matters, which all four strongly affirm, and more about terminology, threshold framing, unit/reporting philosophy, and how Lp(a) is operationalized in risk assessment pathways [10,12,13,14]. A practical application of this approach is summarized in Figure 4 and Table 2.

## 6. Interpretation and Measurement Principles

Across current guidelines, Lp(a) should be viewed as a continuous risk marker, not simply “normal vs. high.” Cardiovascular risk increases progressively with higher levels and is influenced by the units reported, assay design, and clinical context [1,12].

### 6.1. Units and Standardization

Guidelines recommend reporting lipoprotein(a) [Lp(a)] in molar units (nmol/L) rather than mass-based units (mg/dL), as the latter are confounded by structural variability in apolipoprotein(a) [apo(a)] [4,16]. In practical terms, nmol/L reflects particle number, whereas mg/dL reflects total mass. This distinction is clinically important because Lp(a) particle size varies across individuals due to differences in kringle IV type 2 repeats within apo(a). As a result, mass-based assays are subject to isoform size bias, where particle size influences measured values independent of particle number. Thus, mg/dL may appear elevated in patients with larger particles despite similar particle numbers and lower in those with smaller particles despite higher particle concentrations. Consequently, mg/dL can overestimate or underestimate true atherogenic exposure [4,16].

Because apo(a) isoform size and assay characteristics influence the relationship between mass and molar concentration, no universal conversion factor between mg/dL and nmol/L should be applied. Where guidelines specify thresholds in both units, these should be interpreted as guideline-defined parallel thresholds rather than exact mathematical equivalents [4].

The 2025 updates from the European Society of Cardiology and European Atherosclerosis Society endorse nmol/L as the preferred unit due to variability across assays, while acknowledging that mg/dL remains acceptable in clinical practice [12]. Similarly, the National Lipid Association emphasizes a continuous risk relationship and provides thresholds primarily in nmol/L, with approximate mg/dL equivalents [13]. Despite progress, assay standardization remains incomplete, underscoring the need for isoform-insensitive assays and International Federation of Clinical Chemistry and Laboratory Medicine/World Health Organization–traceable reference standards [4,16].

### 6.2. Testing Frequency

Most guidelines recommend measuring lipoprotein(a) [Lp(a)] once in adulthood, as levels are largely genetically determined and relatively stable over time. Routine repeat testing is not required but may be considered in select situations, such as post-menopause, renal, hepatic, or thyroid disease, pregnancy, or systemic inflammation, where levels or their interpretation may change, particularly if prior values were borderline. Lp(a) may also exhibit modest acute-phase behavior, with transient increases during acute illnesses such as acute coronary syndrome or stroke; therefore, measurements obtained during these settings should be interpreted with caution [10,12].

## 7. Risk Stratification Pathways

The 2026 ACC/AHA multi-society dyslipidemia guideline supports a layered approach to cardiovascular risk assessment that integrates baseline risk estimation using PREVENT, personalization with risk-enhancing factors such as Lp(a), and selective coronary artery calcium (CAC) scoring when treatment decisions remain uncertain [10,11]. PREVENT categorizes 10-year ASCVD risk as low (<3%), borderline (3–<5%), intermediate (5–<10%), or high (≥10%). Within this framework, elevated Lp(a) identifies inherited risk not fully captured by conventional risk factors, whereas CAC provides direct evidence of subclinical coronary atherosclerosis and can further refine treatment decisions. Together, these complementary tools support a more individualized approach to preventive therapy [10,11] [Figure 4].

In appropriately selected primary-prevention patients for whom treatment decisions remain uncertain, CAC scoring may further refine risk. The 2026 ACC/AHA guideline recommends consideration of both absolute CAC burden and age-, sex-, and race-standardized percentiles. CAC 1–99 with a value below the 75th percentile generally supports an LDL-C goal of <100 mg/dL, whereas CAC ≥100–299 or CAC ≥75th percentile supports more intensive LDL-C lowering toward <70 mg/dL. CAC 300–999 represents a high subclinical atherosclerotic burden for which substantial LDL-C reduction is warranted, and CAC ≥1000 identifies an especially high-risk group for whom an LDL-C goal <55 mg/dL is recommended. Importantly, CAC = 0 may lower near-term risk in selected individuals but does not eliminate lifetime risk associated with genetically elevated Lp(a), particularly in younger adults.

Incorporating Lp(a) into conventional cardiovascular risk-prediction models generally produces only modest improvement in overall discrimination, although its effect on risk reclassification may be more clinically relevant in selected individuals near treatment thresholds. For example, one recent externally validated model demonstrated only a small increase in C-statistic after adding Lp(a) (0.7475 to 0.7556 in the derivation cohort and 0.7350 to 0.7368 in external validation), while net reclassification improvement among individuals at borderline or intermediate risk was 21.3%. These estimates are model- and population-specific and should not be interpreted as coefficients for mathematically recalibrating PREVENT-ASCVD risk [13,14]. Several tools have been developed to illustrate the incremental cardiovascular risk attributable to elevated Lp(a). The Lp(a) Clinical Guidance calculator, developed in association with the EAS consensus framework, estimates cardiovascular risk both before and after accounting for Lp(a), thereby demonstrating the additional risk associated with a given Lp(a) concentration. Importantly, the calculator also models the potential reduction in overall cardiovascular risk achieved through modification of other treatable risk factors, particularly LDL-C and blood pressure. This provides a practical framework for demonstrating how more intensive management of modifiable risk factors may partially mitigate the excess risk associated with elevated Lp(a), even when Lp(a) itself remains unchanged [10,11,13,14,17] (Table 3).

### Sex-Specific Considerations

Sex-related differences are increasingly recognized as important components of cardiovascular risk assessment. Although Lp(a) concentrations are predominantly genetically determined, levels may increase after menopause, and the clinical expression of cardiovascular risk occurs within a broader risk profile that differs between women and men. Nevertheless, current evidence does not support separate sex-specific Lp(a) thresholds, and contemporary guidelines do not recommend different definitions of elevated Lp(a) according to sex. Accordingly, sex should be incorporated into global cardiovascular risk assessment rather than used to modify Lp(a) thresholds independently. Further research is needed to determine whether interactions among Lp(a), menopause, sex-specific risk factors, and imaging-defined subclinical atherosclerosis should influence future risk-refinement strategies [19].

## 8. Clinical Management Recommendations

### Therapeutic Approaches for Elevated Lp(a)

Management of elevated lipoprotein(a) [Lp(a)] remains challenging, as there are currently no approved therapies specifically targeting Lp(a) [1]. Because Lp(a) levels are largely genetically determined, lifestyle interventions such as diet and exercise have minimal effect. However, comprehensive cardiovascular risk reduction, including a healthy diet, smoking cessation, and optimal blood pressure, remains essential.

Current management therefore focuses on aggressive control of modifiable ASCVD risk factors, particularly [10,11]:

- Intensive LDL-C lowering
- Optimization of blood pressure, glycemic control, and lifestyle factors
- Selective use of coronary artery calcium (CAC) to refine risk

In patients with elevated Lp(a), clinicians should adopt a more intensive preventive strategy, even in the absence of Lp(a)-specific therapies [1,10,12]. Consistent with the 2024 Polish Cardiac Society and Polish Lipid Association recommendations, identification of elevated Lp(a) should prompt reassessment of overall cardiovascular risk and optimization of modifiable risk factors rather than treatment of the Lp(a) concentration in isolation. In selected primary-prevention patients, CAC assessment may further refine risk, while progressively more intensive LDL-C–lowering strategies should be considered according to overall cardiovascular risk. Lipoprotein apheresis may be considered in highly selected patients with progressive cardiovascular disease despite optimal control of other risk factors and markedly elevated Lp(a) [20].

1. Lipid-Lowering Therapies:

Most conventional lipid-lowering therapies have little to no direct effect on Lp(a) and are used primarily to reduce overall cardiovascular risk.

2. Statins: Statins do not meaningfully reduce Lp(a) and may cause modest increases. However, they remain the foundation of therapy due to their proven benefit in reducing ASCVD risk. Elevated Lp(a) should prompt more intensive statin use, not avoidance [10,21].
3. Niacin: Niacin can reduce Lp(a) by approximately 20–30%, but randomized trials have not demonstrated cardiovascular benefit when added to statin therapy. Due to lack of outcome benefit and adverse effects, it is not recommended solely for Lp(a) reduction [22,23].
4. Ezetimibe and fibrates: Ezetimibe and fibrates have minimal or negligible effects on Lp(a). Their use is directed at managing LDL-C and other lipid abnormalities rather than Lp(a) [10,12].
5. PCSK9 Inhibitors: Proprotein convertase subtilisin/kexin type 9 inhibitors (e.g., alirocumab, evolocumab) are injectable cholesterol-lowering drugs that incidentally lower Lp(a) levels by about 20–30% [24,25]. Although the mechanism of Lp(a) reduction is not entirely clear, it is proposed that the increased clearance of LDL receptor recycling indirectly clears Lp(a) particles from circulation. In major trials such as ODYSSEY Outcomes and FOURIER, Lp(a) reduction was associated with improved cardiovascular outcomes, particularly in patients with higher baseline Lp(a) [24,26]. While these findings suggest that Lp(a) lowering may contribute to benefit, this relationship remains observational. PCSK9 inhibitors modestly lower Lp(a), typically by approximately 20–30%; however, they are not approved or recommended specifically for isolated Lp(a) lowering. Their use should follow established indications for LDL-C lowering and cardiovascular risk reduction, including appropriate patients with ASCVD, familial hypercholesterolemia, or persistent LDL-C elevation despite maximally tolerated therapy. Elevated Lp(a) may contribute to overall risk assessment but should not independently determine PCSK9 inhibitor initiation [10,24,25].
6. Aspirin: Elevated Lp(a) has prothrombotic and antifibrinolytic properties, raising interest in whether antiplatelet therapy may provide additional benefit in selected individuals. In observational primary-prevention data, aspirin use has been associated with a lower risk of coronary heart disease among individuals with elevated Lp(a), including an approximately 46% lower risk among participants with Lp(a) > 50 mg/dL in the Multi-Ethnic Study of Atherosclerosis. More recently, an exploratory MESA analysis found that regular aspirin use was associated with lower risks of incident aortic valve calcification and severe aortic stenosis among individuals with high Lp(a). However, these findings remain observational and require confirmation in randomized studies. Elevated Lp(a) alone is therefore not currently an established indication for aspirin; its use should be individualized according to overall ASCVD risk, indication for primary or secondary prevention, and bleeding risk [26].
7. Lipoprotein apheresis: Lipoprotein apheresis is a specialized therapy that can acutely reduce Lp(a) by 50–75% per session, though levels rebound between treatments. Because Lp(a) concentrations rebound between apheresis sessions, time-averaged lipoprotein concentrations may be estimated using the Kroon approach rather than relying solely on immediate pre- or post-apheresis measurements. Lipoprotein apheresis is a selective extracorporeal filtration process that removes apoB-containing lipoproteins from the blood [12]. Since both LDL and Lp(a) contain apoB-100, they are both effectively removed during treatment. In select countries, lipoprotein apheresis is used for patients with markedly elevated Lp(a) and progressive ASCVD despite maximal therapy [12]. Germany permits use for Lp(a) > 60 mg/dL with progressive ASCVD, whereas U.S. FDA indications are narrower, generally limited to patients with familial hypercholesterolemia, established CAD or PAD, LDL-C > 100 mg/dL, and Lp(a) > 60 mg/dL. Apheresis reduces LDL-C and Lp(a) by ~50–75% per session, but levels rebound, requiring weekly or biweekly treatments. Given its invasive, costly nature and limited availability, it is reserved for highly selected, refractory patients [27,28].

## 9. Emerging Lp(a)-Targeted Therapies

### 9.1. Antisense Oligonucleotide Therapy

Targeted Lp(a) lowering has advanced with the development of antisense oligonucleotides (ASOs) designed to inhibit hepatic production of apolipoprotein(a) [29]. Pelacarsen is an ASO that binds apo(a) mRNA in the liver, thereby reducing Lp(a) synthesis. In a phase 2 trial, pelacarsen produced dose-dependent reductions in Lp(a) of up to approximately 80% and was generally well tolerated over 6–12 months of treatment [30]. Substantial dose-dependent absolute reductions in Lp(a) were observed across dosing groups, with many patients achieving markedly lower levels on higher doses [30]. The ongoing phase 3 Lp(a)HORIZON trial is evaluating whether pelacarsen reduces major adverse cardiovascular events (MACE) in patients with established cardiovascular disease [30]. The ongoing phase 3 Lp(a)HORIZON trial is evaluating whether pelacarsen-mediated Lp(a) lowering reduces major adverse cardiovascular events in patients with established cardiovascular disease [30]. Until cardiovascular outcomes data are available, the clinical benefit of pharmacologic Lp(a) lowering remains uncertain. Additional studies, including the Lp(a)FRONTIERS trial, are evaluating its potential role in conditions such as calcific aortic valve stenosis (CAVS) [29,31].

### 9.2. siRNA Therapies

Small interfering RNA (siRNA) is another gene-silencing approach being applied to Lp(a) [32,33,34,35,36].

#### 9.2.1. Olpasiran

Olpasiran is an siRNA targeting hepatic LPA expression. In the phase 2 OCEAN(a)-DOSE trial, olpasiran produced dose-dependent and sustained reductions in Lp(a), exceeding 95% with higher-dose regimens. Injection-site reactions were among the most commonly reported adverse effects. The ongoing phase 3 OCEAN(a)-Outcomes trial is evaluating whether this marked biochemical reduction translates into fewer major cardiovascular events [32].

#### 9.2.2. Other siRNAs

Other siRNA agents, including SLN360, lepodisiran, and zerlasiran, have also shown large Lp(a) reductions in early- or mid-phase studies. SLN360, lepodisiran, and zerlasiran have shown >80–90% Lp(a) reductions [32,33,34,35,36]. The Phase 3 ACCLAIM-Lp(a) Outcomes trial is currently ongoing to study cardiovascular events when used for secondary prevention [32,33,34].

Collectively, these agents demonstrate that substantial and sustained pharmacologic reduction of Lp(a) is feasible. However, the clinical significance of these reductions remains to be established, and ongoing randomized outcomes trials will determine whether Lp(a) lowering translates into fewer cardiovascular events.

### 9.3. Oral Small Molecules

Muvalaplin is an oral small-molecule inhibitor of Lp(a) particle assembly. In phase 2 testing, it produced dose-dependent Lp(a) reductions, with reductions up to 85% using an intact Lp(a) assay and up to 70% using an apo(a)-based assay. This assay-dependent difference is important when interpreting results [37].

Pelacarsen’s Lp(a)HORIZON is a phase 3 cardiovascular outcomes study in established CVD with elevated Lp(a); OCEAN(a)-Outcomes evaluates olpasiran versus placebo for CHD death, MI, or urgent coronary revascularization; ACCLAIM-Lp(a) is evaluating lepodisiran for cardiovascular risk reduction [38].

Several limitations should be considered when interpreting the current Lp(a) literature. Measurement remains affected by variability in assay methodology, apolipoprotein(a) isoform sensitivity, and reporting in either nmol/L or mg/dL, limiting direct comparison across studies and clinical laboratories. In addition, professional societies use differing thresholds and terminology to categorize elevated Lp(a), although cardiovascular risk appears to increase continuously across the concentration spectrum. Most importantly, while emerging Lp(a)-targeted therapies have produced substantial reductions in circulating Lp(a), completed randomized cardiovascular outcomes trials demonstrating that selective Lp(a) lowering reduces clinical events are not yet available. Accordingly, the magnitude of Lp(a) reduction observed in early- and mid-phase trials should not yet be interpreted as evidence of cardiovascular benefit.

Several additional Lp(a)-targeted compounds are in the early stages of clinical development. Given the limited availability of peer-reviewed efficacy data for these programs, they are not included in Table 4; their clinical relevance will require assessment as results from ongoing studies become available.

## 10. Conclusions

Lipoprotein(a) [Lp(a)] has evolved from an underrecognized biomarker to a central determinant of cardiovascular risk, supported by strong genetic, epidemiologic, and mechanistic evidence linking it to atherosclerotic cardiovascular disease (ASCVD) and calcific aortic valve stenosis [1,2,6,7,39]. Contemporary guidelines now endorse once-in-a-lifetime measurement and emphasize its role as a risk-enhancing factor within structured risk assessment frameworks. The 2026 multi-society dyslipidemia guideline reflects a paradigm shift toward layered and individualized risk assessment, integrating baseline risk estimation (PREVENT), genetic risk modifiers such as Lp(a), and imaging-based reclassification using coronary artery calcium (CAC) [10,11,13]. This approach enables more precise identification of high-risk individuals and supports earlier and more intensive preventive strategies. Despite its clear role in risk stratification, management remains indirect, centered on aggressive modification of traditional risk factors, particularly LDL-C lowering, with selective use of PCSK9 inhibitors and lipoprotein apheresis in high-risk patients [10,11].

Novel therapies targeting Lp(a), including antisense oligonucleotides and small interfering RNA agents, have demonstrated substantial and sustained reductions in circulating Lp(a). However, reduction in a biomarker should not be assumed to confer clinical benefit. Ongoing phase 3 cardiovascular outcome trials will determine whether selective Lp(a) lowering reduces cardiovascular events and, consequently, whether Lp(a) can be established as an actionable therapeutic target.

## 11. Future Directions

The central question moving forward is not whether Lp(a) is causal, as this has been firmly established, but whether targeted reduction improves clinical outcomes. Results from ongoing phase 3 trials are expected to clarify this question and will likely determine the future role of Lp(a)-directed therapies in routine practice. In the interim, the priority for clinicians is clear: identify patients with elevated Lp(a) and incorporate this information into risk stratification and management decisions. Given the development of several highly effective Lp(a)-lowering agents, early identification may become increasingly relevant if ongoing outcome trials demonstrate clinical benefit, and therapies subsequently receive regulatory approval. As the field transitions from risk recognition to targeted intervention, Lp(a) may increasingly become a key component of precision cardiovascular prevention, bridging genetic risk, imaging, and individualized therapy.

## Figures and Tables

**Table 1:** Comparing features and structural differences between LDL and Lp(a).

Feature	LDL	Lp(a)
Core lipoprotein	LDL particle	LDL particle
Primary protein	ApoB-100	ApoB-100 + Apo(a)
Linkage		Apo(a)-ApoB-100 disulfide bond link
Size	Relatively uniform	Highly variable (depending on Apo(a) isoforms)
Oxidized phospholipids	Minimal	Enriched
Genetic determination	Moderate	Very high
Response to lifestyle	Significant	Minimal

**Table 2:** Contemporary guideline recommendations (2018–2026).

Guideline/Organization	Year	Screening Recommendation	Target Population	Risk Thresholds	Comments
American College of Cardiology/American Heart Association (Multi-society Dyslipidemia Guideline)	2026	Measure Lp(a) at least once in all adults	All adults; especially useful in borderline/intermediate risk, family history of premature ASCVD, or unexplained events	Risk-enhancing: ≥50 mg/dL (≥125 nmol/L); Major risk enhancer: ≥100 mg/dL (≥250 nmol/L)	Integrated with PREVENT risk model and CAC; Lp(a) used to up-classify risk and guide earlier/intensified therapy
ESC/EAS Focused Update of Dyslipidaemias Guideline	2025	Consider measuring Lp(a) at least once in each adult’s lifetime	All adults, especially those at moderate risk or near treatment-decision thresholds where risk refinement may change management	>50 mg/dL (≈105 nmol/L) considered a CV risk-enhancing factor	Used as a risk modifier to refine CV risk estimation and potentially reclassify patients to a higher-risk category; higher levels imply greater risk.
National Lipid Association	2024 update	Measure Lp(a) at least once in every adult	All adults; especially premature ASCVD, family history, unexplained ASCVD	Low: <30 mg/dL; Intermediate: 30–50 mg/dL; High: ≥50 mg/dL	Risk-enhancing factor; supports cascade screening in families
European Atherosclerosis Society	2022 consensus	Measure at least once in adulthood	All adults; emphasis on FH, premature ASCVD, aortic stenosis	Rule-out: <30 mg/dL; Gray zone: 30–50 mg/dL; Rule-in: >50 mg/dL	Continuous risk; thresholds are pragmatic, not absolute
Canadian Cardiovascular Society	2021 guideline	Once-in-a-lifetime measurement as part of lipid screening	Adults undergoing risk assessment; especially family history/premature ASCVD	High risk: ≥50 mg/dL (≥100 nmol/L)	Used as risk modifier to intensify therapy
American College of Cardiology/American Heart Association (Cholesterol Guideline)	2018	Selective measurement (not universal)	Borderline/intermediate risk; family history of premature ASCVD	Risk-enhancing: ≥50 mg/dL (≥125 nmol/L)	Not included in pooled cohort equations; earlier conservative stance

Data derived from references [1,10,11,12,13,14,15,16].

**Table 3:** Integrated Risk Assessment Framework (PREVENT-Lp(a)-CAC).

Component	Description	Key Details	Clinical Implication
PREVENT risk equation	Baseline 10-year ASCVD risk estimation	Risk categories: Low (<3%), Borderline (3–<5%), Intermediate (5–<10%), High (≥10%)	Establishes initial risk stratification using traditional clinical variables
Lp(a) as a risk enhancer	Inherited risk-enhancing factor considered after baseline risk estimation	Lp(a) ≥125 nmol/L or ≥50 mg/dL is considered a risk-enhancing factor; very high levels are associated with progressively greater long-term ASCVD risk	Supports clinician–patient discussion and may favor earlier or more intensive management of LDL-C and other modifiable risk factors; not a validated mathematical recalibration of PREVENT risk
Coronary Artery Calcium Score Integration (CPR Model)	Imaging-based risk refinement after Lp(a) assessment	CAC and Lp(a) are independent predictors of ASCVD;	Used selectively when risk remains uncertain after PREVENT + Lp(a)
CAC-Guided Management	Use of CAC to guide treatment intensity and targets	High CAC (especially with elevated Lp(a)) → lower LDL-C/non-HDL-C targets	Moves beyond statin intensity alone → goal-directed lipid lowering

Data derived from references [10,11,17,18,19].

**Table 4:** Investigational therapies targeting lipoprotein(a) (Lp[a]).

Drug	Modality	Mechanism/Target	Route and Dosing	Clinical Phase	Key Trial(s)	Approx. Lp(a) Reduction ^†^
Pelacarsen (formerly AKCEA-APO(a)-LRx/IONIS-APO(a)-LRx; TQJ230)	Antisense oligonucleotide	Binds LPA/apo(a) mRNA to reduce hepatic apo(a) production	SC; weekly to monthly regimens studied	Phase 3	NCT03070782; Lp(a)HORIZON(NCT04023 552); Lp(a)FRONTIERS Apheresis (NCT05305664)	Up to ~80% in phase 2
Olpasiran (AMG 890)	siRNA	Silences LPA expression	SC; q12-week regimens prominently studied	Phase 3	OCEAN(a)-DOSE(NCT04270760); OCEAN(a)-Outcomes (NCT05581303); OCEAN(a)-PreEvent(NCT07136012)	>95% at higher phase 2 doses
Zerlasiran (SLN360)	siRNA	Silences LPA expression	SC; infrequent dosing	Phase 2	NCT04606602; ALPACAR-360(NCT05537571)	>80% time-averaged reduction; reports of >90% at later follow-up
Lepodisiran (LY3819469)	siRNA	Silences LPA expression	SC; single-dose and infrequent regimens studied	Phase 3	NCT04914546; ALPACA(NCT05565742); ACCLAIM-Lp(a)(NCT06292013)	~94–95%
Muvalaplin (LY3473329)	Small molecule	Inhibits apo(a)-apoB binding, preventing Lp(a) particle assembly	Oral, once daily	Phase 3 program initiated	NCT04472676; KRAKEN(NCT05563246); MOVE-Lp(a)(NCT07157774)	~70% by apo(a) assay; up to ~85.8% by intact-particle assay

^†^ Reported percentage reductions in Lp(a) are derived from individual clinical trials and should not be directly compared across agents because assay methodology, baseline Lp(a) concentrations, dose, treatment duration, and reported estimates differ. For muvalaplin, reductions differed according to assay methodology, with approximately 70% reduction using an apo(a)-based assay and up to approximately 85% using an intact-particle assay. Data derived from references [30,32,34,35,36,37,38] and ClinicalTrials.gov records.

## Data Availability

No new data was created or analyzed in this study. Data sharing is not applicable to this article.
